# ATKB-PID: an adaptive control method for micro tension under complex hot rolling conditions

**DOI:** 10.1038/s41598-025-85960-w

**Published:** 2025-01-15

**Authors:** Xinkai Xu, Xiangrong Song, Zheng Qi

**Affiliations:** 1 State Key Laboratory of Metallurgical Intelligent Manufacturing System, Beijing, 100071 China; 2 Steel Industry Green and Intelligent Manufacturing Technology Center, China Iron and Steel Research Institute Group, Beijing, 100081 China; 3 Automation Research and Design Institute of Metallurgical Industry Co., Ltd., Beijing, 100081 China

**Keywords:** Electrical and electronic engineering, Materials science, Mathematics and computing

## Abstract

At present, the parameters of the controllers in hot rolling roughing microtension control systems are not adaptively adjustable to variations in working conditions, which compromises both width accuracy and production stability. To address this issue, this paper introduces an ATKB-PID adaptive micro tension control method. This method incorporates a linear attention layer and utilizes a K-Nearest Neighbors (KNN) algorithm to predict the optimal learning rate and inertia coefficient under actual operating conditions. Furthermore, an objective function is tailored to production indices to enhance model performance. Comparative experiments with both established and recently introduced controllers demonstrate that the proposed ATKB-PID method exhibits a smaller steady-state error and quicker adjustment time. The ATKB-PID control method is well-suited for the complex and dynamic microtension control demands in thermal roughing processes, showing promising application potential.

## Introduction

In the steel making process, micro tension control in the roughing mill of hot rolling has a direct effect on plate width accuracy and production stability^1^. This control effect will further affect the finishing process and thus the overall quality of the hot rolled coil^2^. At present, the hot rolling roughing mill micro-tension control mostly relies on empirically presetting PID controller parameters, which have many shortcomings^3^. Production involves fine-tuning the main drive speed of the vertical roller mill motor according to the difference between the actual tension between the vertical and flat rolls and the theoretical tension to achieve micro-tension control^4^. The target speed and load of the vertical roller mill motor change frequently, resulting in fixed PID parameters, making it difficult to achieve the desired control effect, which affects the precision of steel plate width and product quality. In addition, manual adjustment of PID parameters is inefficient and greatly reduces the stability and efficiency of production^5^.

At present, there are two main solutions to optimize the micro-tension control: the first is a more accurate prediction of the PID parameters^6^, some scholars have improved the effect of micro-tension control by improving the mechanism model^7^, but still can not adapt to the complexity of working conditions on the site; the second is the design of adaptive controllers, the most typical adaptive controller is the BP-PID^8^, which can be adapted to adjust the parameters of the controller for different conditions to improve the stability and efficiency of production and meet the production stability and product quality requirements. Improve the stability and efficiency of production to meet the technical requirements. At present, BP-PID has been widely used in various industrial fields, Ren et al. proposed an active anti-disturbance pitch controller using BP-PID algorithm for different working conditions of wind turbines to meet the engineering needs^9^, and BP-PID has also been applied to the lateral stability control of electric vehicles^10^, DC electronic load systems^11^ and so on. However, its control performance is affected by two factors: the parameter settings (learning rate and inertia coefficient)^12^ and the susceptibility of the BP algorithm to gradient vanishing^13^. Adaptive controllers with extremely irrational parameter settings can adapt to different operating conditions, but it will lead to its long control time, so predicting roughly reasonable parameters through different operating conditions can effectively shorten the control time of the BP-PID controller^14^, and Adithiyaa et al. have already proved the ability of KNN to predict the parameters^15^; and Wang et al. have proposed to introduce the attention mechanism into the BP algorithm to help update the weight matrix according to the inputs, which effectively solves the problem that the BP algorithm itself is prone to gradient vanishing^16^. Moreover, the objective function has been shown to have a significant effect on the steady-state error, overshoot amount, control time and other indices in adaptive control tasks^17^. Considering that the controlled object is discrete and has a large time step, the system is a black box, and the PID controller in the hot rolling system is difficult to replace, it is best to make improvements based on the PID controller.

Therefore, we propose a novel adaptive micro tension control method specifically applied to the hot rolling roughing process. Firstly, we introduce a linear attention mechanism, which is more suitable for fast response scenarios compared to ordinary attention mechanisms, to optimize the BP neural network to avoid its potential gradient vanishing problem. Secondly, we adopt the KNN algorithm to achieve the learning rate and inertia coefficient of the adaptive controller parameters according to the working conditions. Finally, we design a specific objective function to improve the performance of the adaptive controller according to the control task to ensure that the control effect of the micro tension control of the hot rolling roughing mill meets the engineering needs^18^. In this paper, this micro-tension control method is referred to as the ATKB-PID micro-tension control method.

## Methods

In this chapter, we introduce how we designed the ATKB-PID controller specifically for the discrete and black-box system of hot continuous rolling.

### The shortcomings of the BP-PID controller

**Fig. 1:**
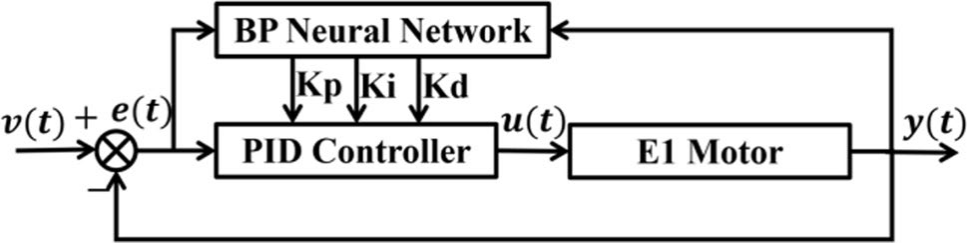
BP-PID controller structure.

The BP neural network’s popularity stems from its capabilities in parallel computing and adaptive learning, operating without the need for detailed system information on nonlinear control objects, relying only on input-output data^11^. The control principle of the BP-PID controller is illustrated in Fig. 1.

Nonetheless, the BP algorithm’s data transition from input to hidden layers lacks information on input value relationships, reducing correlation and potentially prolonging adaptive control, thus impacting controller performance^19^.

### Controller incorporating Linear attention

Attention has become a key technique in the field of deep learning in recent years, and the attention mechanism can automatically focus on important information while ignoring irrelevant information when processing data^20^. Introducing an attention mechanism into the controller allows it to extract deeper information without changing the information’s dimension, thereby enhancing the adaptive controller’s performance. Traditional attention mechanisms are slower in inference due to the softmax function. In contrast, linear attention mechanisms, which do not use softmax classification, offer faster inference speeds and are better suited for fast-response adaptive control scenarios^21^. The input layer of the BP neural network connects to the attention layer, where the corresponding weights are determined by calculating attention scores using the softmax function^22^.

**Fig. 2:**
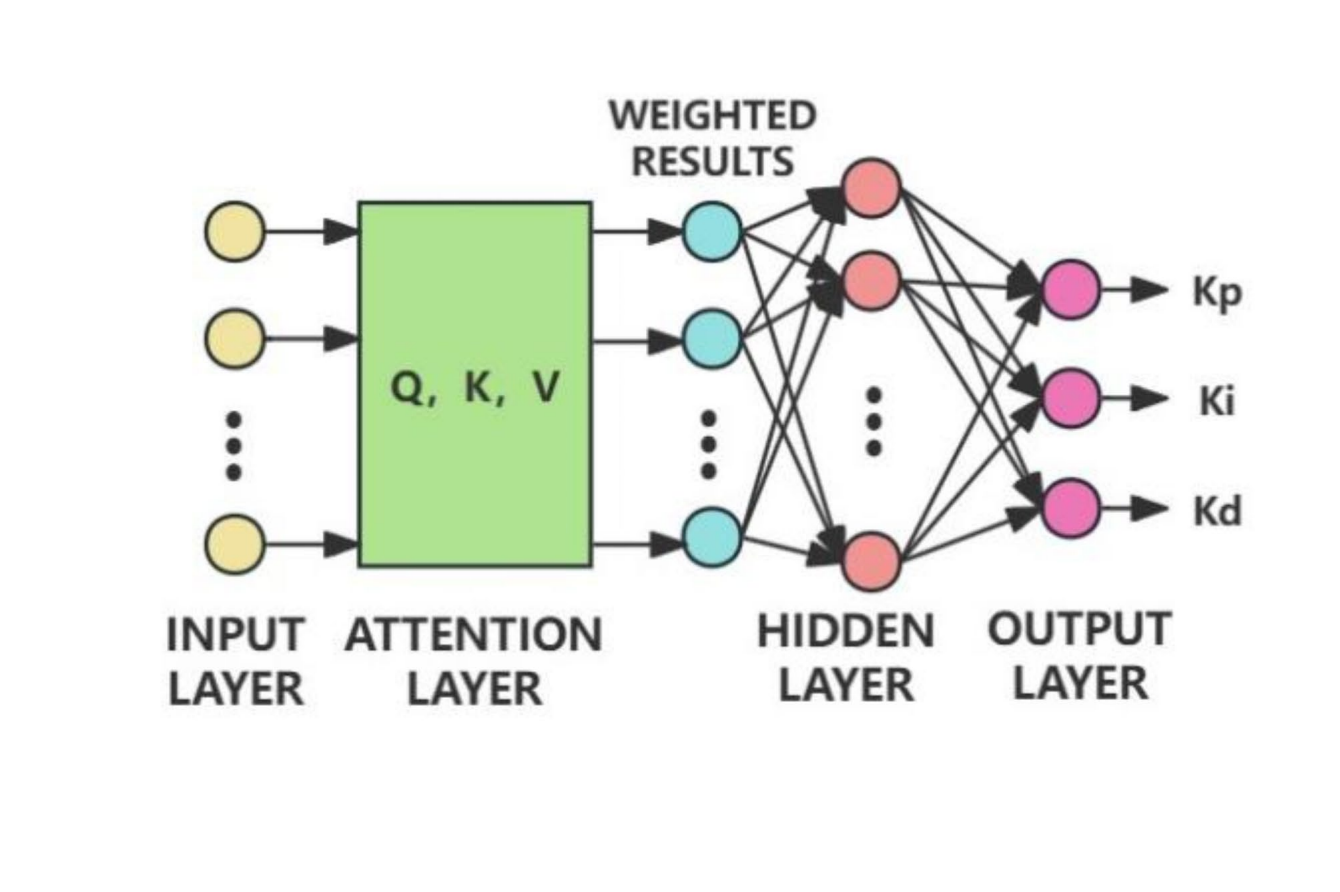
BPNN structure with the addition of a linear attention layer.

These weights, derived from attention scores and the softmax function, are then used to calculate the weighted vectors that are input into the hidden layer of the BP neural network. The specific network structure is shown in Fig. 2, and the traditional attention mechanism can be mathematically represented as Eq. 1.

1 $$\:Attention(Q,K,V)=softmax(Q\bullet\:{K}^{T})V$$

In Eq. 1, $\:Q\bullet\:{K}^{T}$ yields a matrix of $\:n\times\:n$, making the complexity of model is $\:O\left({n}^{2}\right)$.

**Fig. 3:**
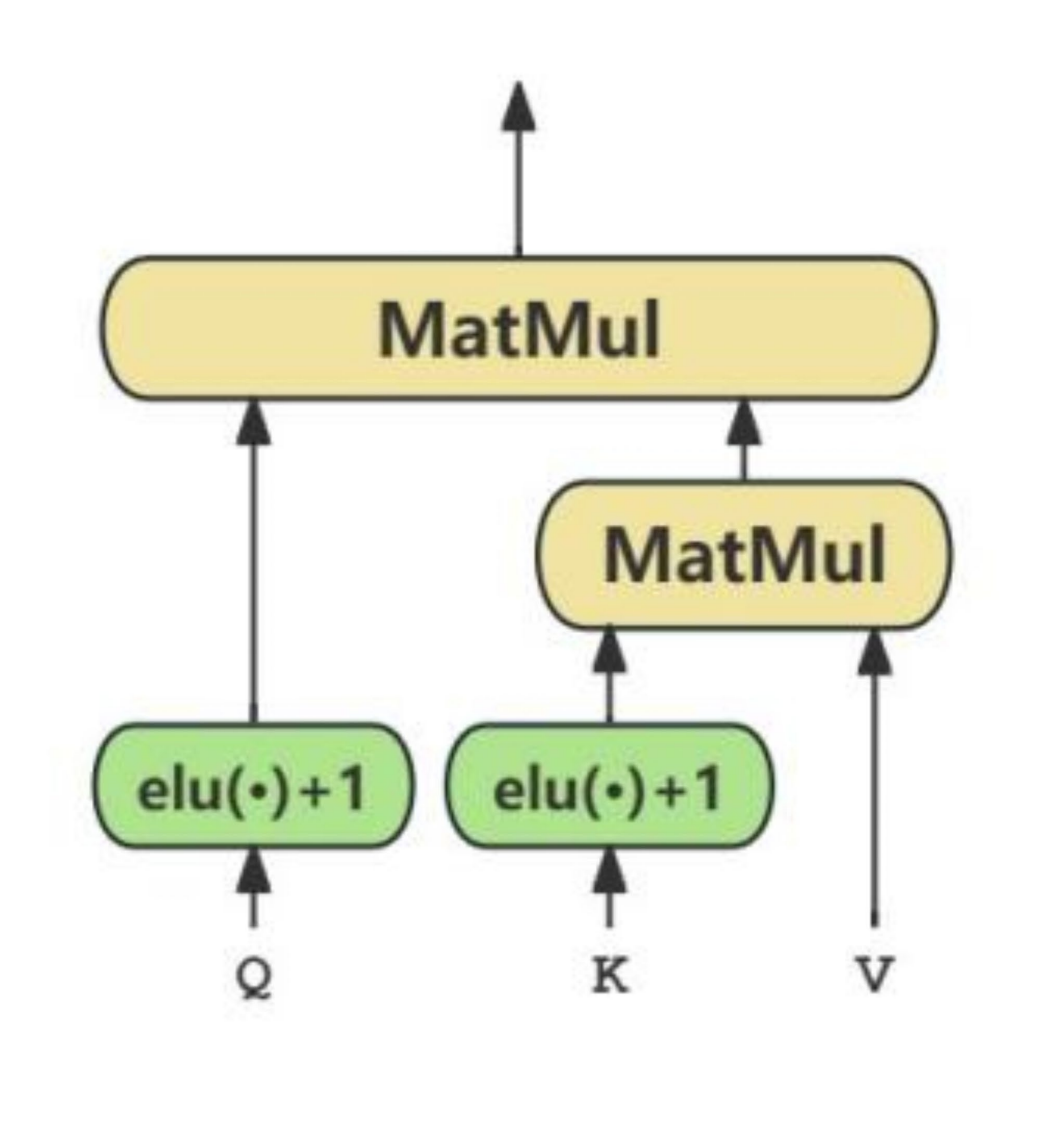
Structure of the linear attention layer.

The structure of the linear attention layer is shown in Fig. 3.

The linear attention mechanism can be mathematically represented as Eq. 2. The complexity of the model using the linear attention is $\:O\left(n\right)$, much less than $\:O\left({n}^{2}\right)$.

2 $$\:LinearAttention(Q,K,V)=Q\bullet\:sim({K}^{T}\bullet\:V)$$

### KNN rectification of BP-PID controller parameters

The ATKB-PID controller uses an incremental PID algorithm. The input $\:{net}_{i}^{\left(2\right)}\left(k\right)$ from attention layer and output $\:{O}_{i}^{\left(2\right)}\left(k\right)$ of the intermediate layer nodes in the ATKB neural network are as shown in Eq. 3:

3 $$\:\left\{\begin{array}{c}{net}_{i}^{\left(2\right)}\left(k\right)=\sum\:_{j=1}^{M}{\omega\:}_{ij}^{\left(2\right)}{x}_{j}^{\left(1\right)}\\\:{O}_{i}^{\left(2\right)}\left(k\right)=f\left({net}_{i}^{\left(2\right)}\left(k\right)\right),i=\text{1,2},\dots\:,Q\end{array}\right.$$

In Eq. 3, $\:{\omega\:}_{ij}^{\left(2\right)}$ is the weighted coefficients between the input layer neurons and the hidden layer neurons, and the function $\:f\left(x\right)=\frac{{e}^{x}-{e}^{-x}}{{e}^{x}+{e}^{-x}}$. Further, the inputs and outputs of the network’s output layer can be obtained as shown in Eq. 4.

4 $$\:\left\{\begin{array}{c}{net}_{l}^{\left(3\right)}\left(k\right)=\sum\:_{j=1}^{P}{\omega\:}_{li}^{\left(3\right)}{O}_{i}^{\left(2\right)}\\\:{O}_{1}^{\left(3\right)}\left(k\right)={k}_{P}\left(k\right)=h\left({net}_{1}^{\left(3\right)}\left(k\right)\right)\\\:{O}_{2}^{\left(3\right)}\left(k\right)={k}_{I}\left(k\right)=h\left({net}_{2}^{\left(3\right)}\left(k\right)\right)\\\:{O}_{3}^{\left(3\right)}\left(k\right)={k}_{D}\left(k\right)=h\left({net}_{3}^{\left(3\right)}\left(k\right)\right)\end{array}\right.$$

In Eq. 4, $\:{\omega\:}_{li}^{\left(3\right)}$ is the weighted coefficients between the hidden layer neurons and the output layer neurons.

The deviation between the system’s actual output and the expected output is denoted as $\:e\left(k\right)={O}_{i}-{x}_{j}$. By employing an incremental PID control algorithm, the value of the PID control quantity $\:u\left(k\right)$ can be calculated. The result of its discretization is shown in Eq. 5:

5 $$\:\left\{\begin{array}{c}u\left(k\right)={k}_{P}\{e\left(k\right)+\frac{T}{{T}_{I}}\sum\:_{i=1}^{k}e\left(i\right)+\frac{{T}_{D}}{T}\left[e\left(k\right)-e\left(k-1\right)\right]\}\\\:u\left(k-1\right)={k}_{P}\{e\left(k-1\right)+\frac{T}{{T}_{I}}\sum\:_{i=1}^{k}e\left(i\right)+\frac{{T}_{D}}{T}\left[e\left(k-1\right)-e\left(k-2\right)\right]\}\end{array}\right.$$

In the Eq. 5, $\:{\text{T}}_{\text{I}}$ and $\:{\text{T}}_{\text{D}}$ represent the integral and derivative time parameters, respectively. Further, the control increment of the PID can be obtained, as shown in Eq. 6:

6 $$\:\varDelta\:u\left(k\right)={k}_{P}\left[e\left(k\right)-e\left(k-1\right)\right]+{k}_{I}e\left(k\right)+{k}_{D}[e\left(k-1\right)-2e\left(k-1\right)+e(k-2\left)\right]$$

The most common objective function for a BP-PID controller is shown in Eq. 7:

7 $$\:E\left(k\right)=\frac{1}{2}{({O}_{i}-{x}_{j})}^{2}$$

The model parameter update formula, derived using the gradient descent method, is shown in Eq. 8:

8 $$\left\{ {\begin{array}{*{20}c} {\omega _{{li}}^{{\left( 3 \right)}} \left( k \right) = \alpha \Delta \omega _{{li}}^{{\left( 3 \right)}} \left( {k - 1} \right) + \eta \delta _{l}^{{\left( 3 \right)}} O_{i}^{{\left( 2 \right)}} \left( k \right)} \\ {\delta _{l}^{{\left( 3 \right)}} = E\left( k \right)sgn\left( {\frac{{\partial y\left( k \right)}}{{\partial \Delta u\left( k \right)}}} \right)\frac{{\partial \Delta u\left( k \right)}}{{\partial O_{l}^{{\left( 3 \right)}} \left( k \right)}}h'\left( {net_{l}^{{\left( 3 \right)}} \left( k \right)} \right)} \\ {\omega _{{ij}}^{{\left( 2 \right)}} \left( k \right) = \alpha \Delta \omega _{{ij}}^{{\left( 2 \right)}} \left( {k - 1} \right) + \eta \delta _{l}^{{\left( 2 \right)}} O_{j}^{{\left( 1 \right)}} \left( k \right)} \\ {\delta _{l}^{{\left( 2 \right)}} = f'\left( {net_{i}^{{\left( 2 \right)}} \left( k \right)} \right)\mathop \sum \limits_{{l = 1}}^{3} \delta _{l}^{{\left( 3 \right)}} \omega _{{li}}^{{\left( 3 \right)}} \left( k \right)} \\ \end{array} } \right.$$

In Eq. 8, ${\text{h}}\left( {\text{x}} \right)$ is a non-negative Sigmoid function: $h\left( x \right) = \frac{{e^{x} }}{{e^{x} + e^{{ - x}} }}$. $h^{\prime } \left( x \right) = h\left( x \right)\left( {1 - h\left( x \right)} \right)$, $\:{f}^{{\prime\:}}\left(x\right)=(1-{f}^{2}\left(x\right))/2$, $\:{\upomega\:}$ is the matrix of the implied layer coefficients, $\:{\upalpha\:}$ is the inertia coefficient, and $\:{\upeta\:}$ is the learning rate.$\:\:{\upalpha\:}$ and $\:{\upeta\:}$ have a more significant effect on the BP-PID controller performance. In this paper, the E1 motor of the vertical mill of the 1500 hot rolling roughing mill is modeled by SIMULINK. The learning rate of the controller is set to 0.3, and the influence of the inertia coefficient on the control effect is verified through experiments.

**Fig. 4:**
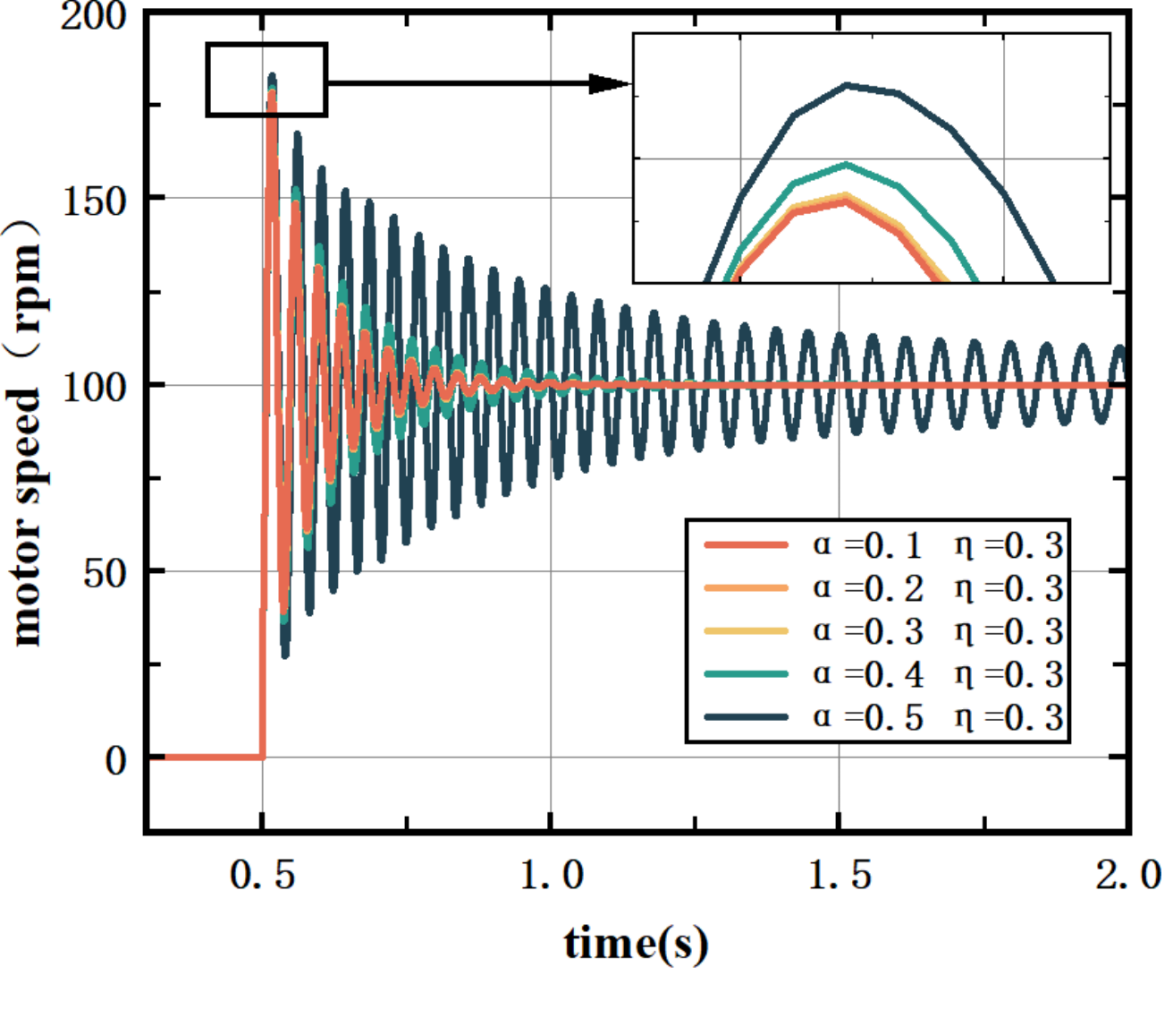
Effect of inertia coefficients on the control effect of the BP‒PID controller.

In Fig. 4, a smaller inertia coefficient increases system sensitivity and speed, resulting in shorter regulation times and smaller overshoots. Conversely, a larger inertia coefficient provides greater damping, enhancing system stability. However, an excessively large inertia coefficient can slow down the BP-PID controller’s response, leading to poor control effects.

**Fig. 5:**
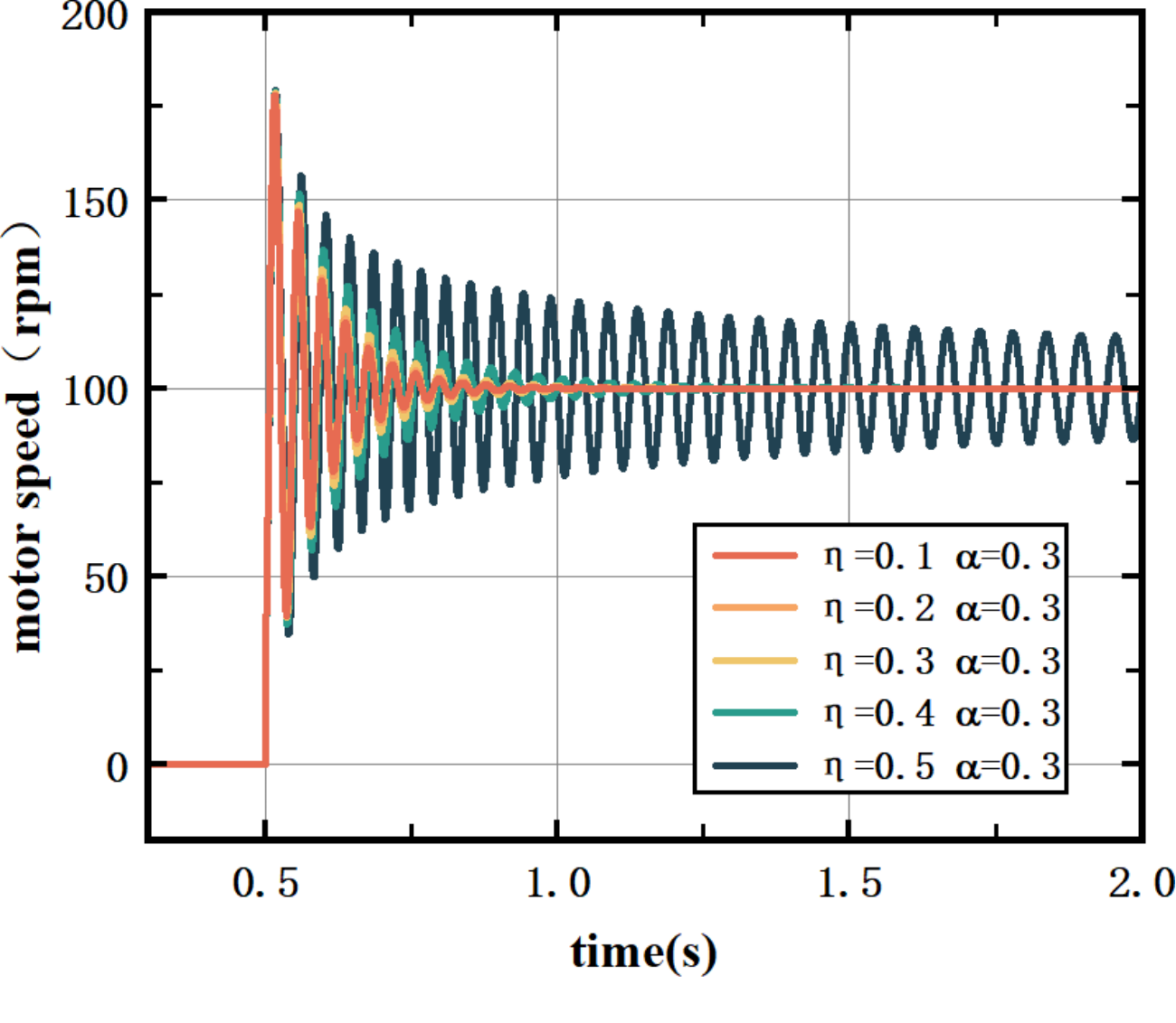
Effect of the learning rate on the control effectiveness of BP-PID controllers.

The target speed of the vertical roller E1 motor is set to 100 rpm, and the motor load is 500 NM, $\:{\upalpha\:}$= 0.3. Experiments are conducted to verify the influence of the learning rate on the control effect of the BP-PID controller. The experiment is shown in Fig. 5.

In Fig. 5, the control effect is best when the learning rate is $\:{\upeta\:}=0.2$, the overshoot is small, the rise time is short, and the regulation time is short; when the learning coefficient is too large, the response of the BP-PID controller is too slow, and the control effect is poor. By adjusting the size of the learning coefficient, the fast response and stability of the system can be balanced.

**Fig. 6:**
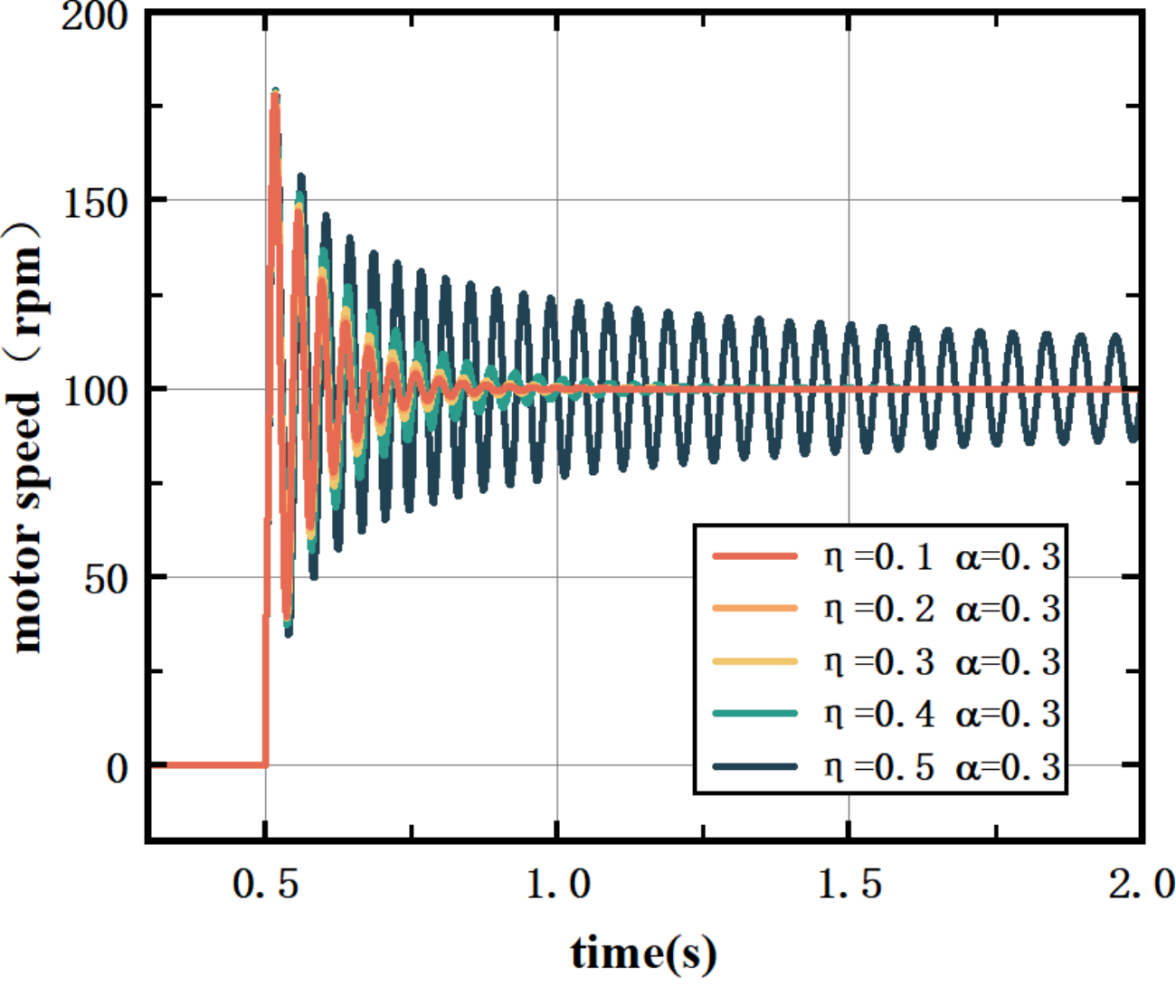
Controller control effect on the E1 motor under different loads.

In summary, the inertia coefficient and the learning coefficient strongly influence the control effect of the BP-PID controller. In the hot rolling roughing process, the speed and load of the E1 motor of the vertical roller mill fluctuate continuously with varying working conditions, thereby increasing the complexity of the controller’s requirements. The control effect of the controller under different loads is tested. The target speed of the motor is set to 100 rpm, and the load is selected from the actual roughing process load range. The experimental results are shown in Fig. 6.

In Fig. 6, increasing the load on the E1 motor results in a significant rise in system rise time, a slight decrease in overshoot, and a marked increase in regulation time. Therefore, different loads at various target speeds can significantly affect the control effect for the E1 motor of the roughing vertical roller mill in the hot rolling process. In this paper, we employ the KNN algorithm to predict the appropriate inertia and learning coefficients for the E1 motor’s target speed and load in the roughing mill. Additionally, we use it to design the objective function.

### ATKB-PID controller

**Fig. 7:**
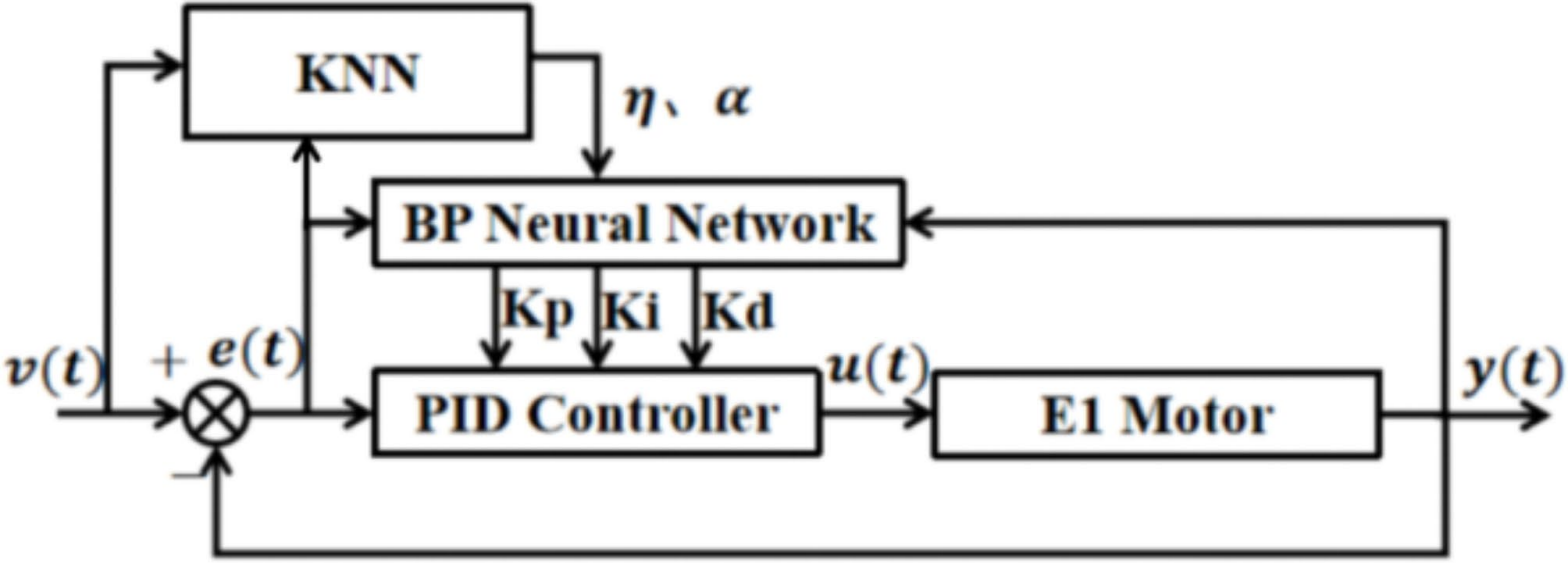
Structure of the ATKB-PID controller.

The control schematic of the modified ATKB-PID controller used in this paper is shown in Fig. 7.

The input parameters for the roughing vertical roller mill E1 include the target motor speed and motor load size, which serve as inputs for the KNN algorithm. The KNN model then predicts the appropriate inertia and learning coefficients for the controller as output through a classification process. The ATKB-PID controller adjusts the inertia and learning coefficients of the BP-PID controller using a machine learning approach. It also enhances and optimizes the objective function. This approach prevents poor control of the E1 motor of the vertical roller mill, which can result from improper parameter settings, making the ATKB-PID controller more adaptable to various working conditions.

### KNN algorithm

KNN is a simple and efficient machine learning classification algorithm that does not need to be trained in advance, and the effect depends on the value of K. It is suitable for rapid prediction of micro-tension control in the roughing process of hot rolling under complex working conditions. When the K value is small, the model is complex and the training error is small, but the generalisation is not good; when the K value is large, the model is simple and the training error is large, but at the same time the generalisation is excellent^14^.

The actual production of E1 vertical roller motor has a variety of working conditions, requiring a variety of load size and motor speed combinations. The selection of the optimal K value for the KNN algorithm was determined through cross-validation, considering various working conditions of the E1 vertical roller motor. Our dataset, comprising 100 samples, covers a wide range of loads and motor speeds encountered in typical hot rolling processes. In practical engineering, the optimal controller parameters of 4–6 working conditions in the data set are the same. Considering that the time complexity of KNN is proportional to the size of K value, as shown in Eq. 9, this paper chooses K value 4.

9 $$\:O\left(K\right)=n\bullet\:K$$

### Objective function

The effect of the objective function on the model is very significant, and a large number of studies have improved the model performance by designing the objective function^23^. In this paper, a new objective function is designed, as shown in Eq. 10, to replace the objective function in Eq. 7.

10 $$\:E\left(k\right)={n}_{1}\bullet\:{\sum\:}_{0}^{k}{e\left(k\right)}^{2}+{{n}_{2}\bullet\:{\sum\:}_{0}^{k}k\bullet\:\left|e\right(k\left)\right|+\:n}_{3}\bullet\:{N}_{\sigma\:}$$

In Eq. 10, $\:e\left(k\right)$ is the absolute error, $\:{\text{N}}_{{\upsigma\:}}$is the number of overshoots, and k is the weighting coefficient. In the micro tension control problem in the roughing region of hot rolling, accuracy, rapidity and overshoot are important indicators of model performance^24^. Therefore, the objective function designed in this paper uses the integral of the absolute error time to improve the model rapidity and accuracy, the integral of the absolute error squared to improve the model accuracy, and the number of overshoots as a penalty term to increase the damping and reduce the number of oscillations^25^. We compared the common objective function with the newly designed objective function when the load was 500NM and the target speed was 100 rpm, as shown in Table 1.

**Table 1:** Control effect with different objective functions.

Objective Function	MSE	MAE	PROPOSED
$\:{N}_{\sigma\:}$	8	8	3
tr	0.035 s	0.039 s	0.039 s
ess	0.05	0.046	0.01

## Results

**Fig. 8:**
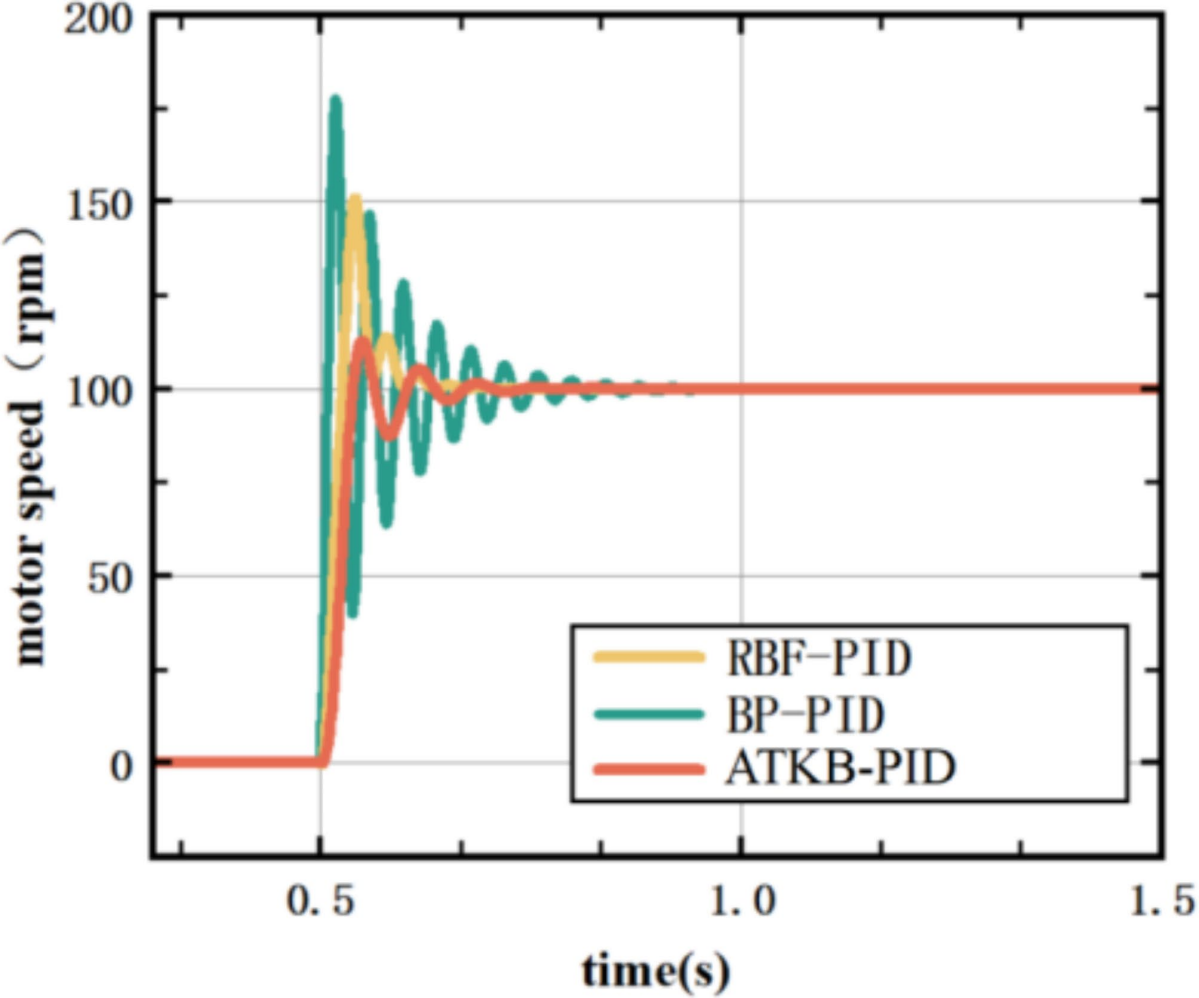
Control effect at a target speed of 100 rpm and a load of 500 NM.

In the experiment, the main configuration of the ATKB-PID controller is as follows: the K value is set to 4, and the initial weight matrices for the hidden layer and the output layer are random numbers ranging from 0 to 0.5. The parameter values of the objective function for $\:{n}_{1}$, $\:{n}_{2}$, $\:{n}_{3}$ are 0.4, 0.4, 0.2. Time step is 0.1s. The vertical roll E1 mill motor is tested at a target speed of 100 rpm and a load of 500 NM, and the results of the experiment are shown in Fig. 8.

In Fig. 8, the ATKB-PID controller outperforms both the ordinary BP-PID and RBF-PID controllers, exhibiting a smaller overshoot. While its rise time is slightly longer than the RBF-PID controller’s, its regulation time is significantly shorter.

**Fig. 9:**
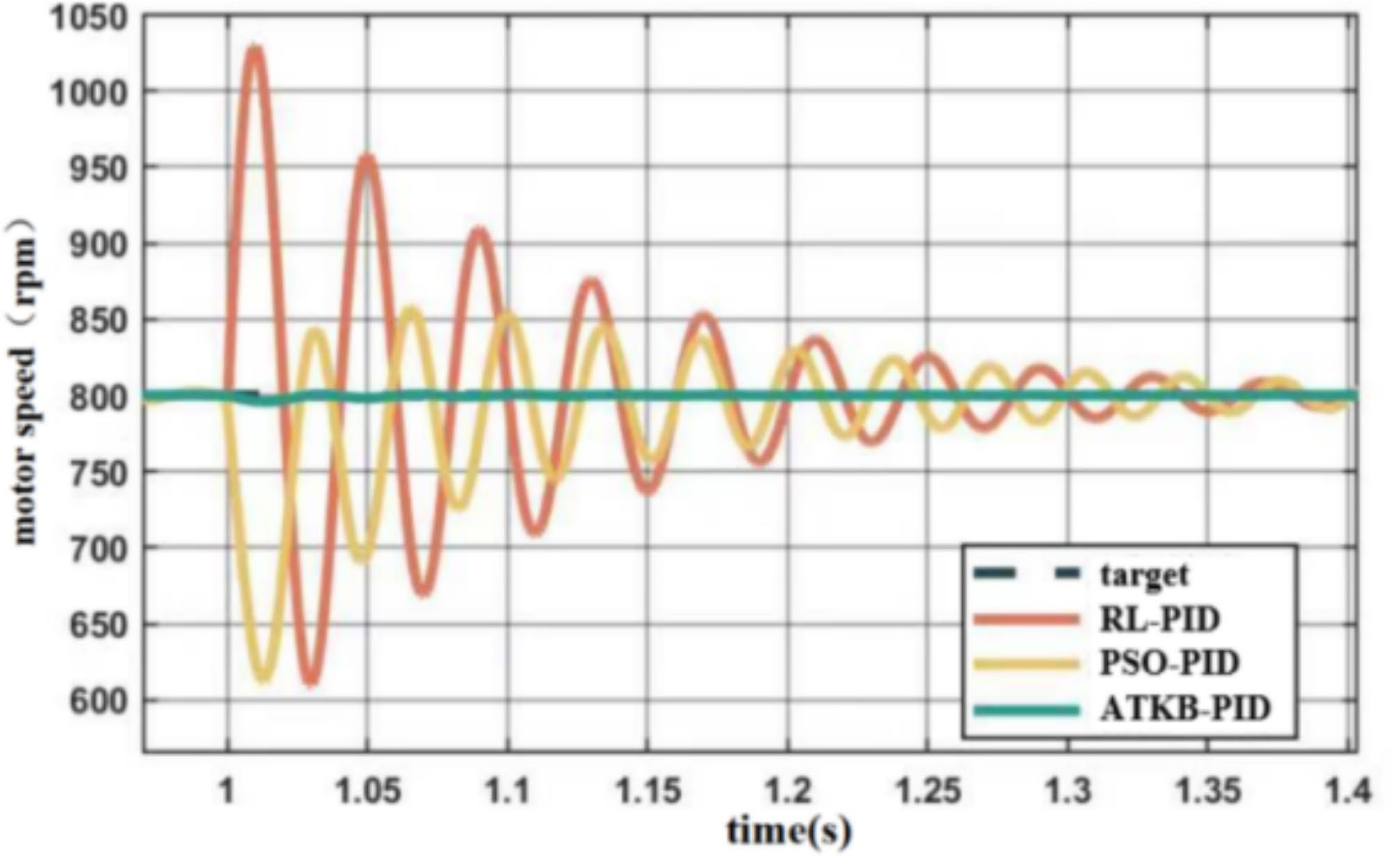
Control effect at the constant speed and the load changes dramatically.

Recently, R. Muduli et al.^26^ proposed a reinforcement learning-based RL-PID controller, suitable for similar working conditions. Experiments were conducted to observe the performance after the mill rolls bite the steel, a point at which the load changes dramatically, and the results are depicted in Fig. 9.

Figure 9shows that during steel rolling, once the mill rolls bite the steel, the ATKB-PID controller demonstrates superior speed regulation compared to both the RL-PID and PSO-PID controllers. Additionally, we incorporated the modified GWO-RL^27^ (Reinforcement Learning and Grey Wolf Optimizer) control method, which is an improved version of GWO-NN, as a comparative method for this task. However, under the initial working conditions, their performance is similar. The relevant experimental parameters of the five controllers are shown in Table 2.

**Table 2:** Control effect with different controllers.

Controller	RBF-PID	BP-PID	RL-PID	PSO-PID	GWO -NN	ATKB-PID
ess	0.03	0.01	0.02	0.01	0.05	0.01
tr	0.011 s	0.030 s	0.035 s	0.030 s	0.050 s	0.039 s
ts	0.284 s	0.476 s	0.260 s	0.307 s	0.608 s	0.268 s

The ATKB-PID controller has a better control effect on different speeds and loads of the E1 motor of the roughing standing roller mill.

**Fig. 10:**
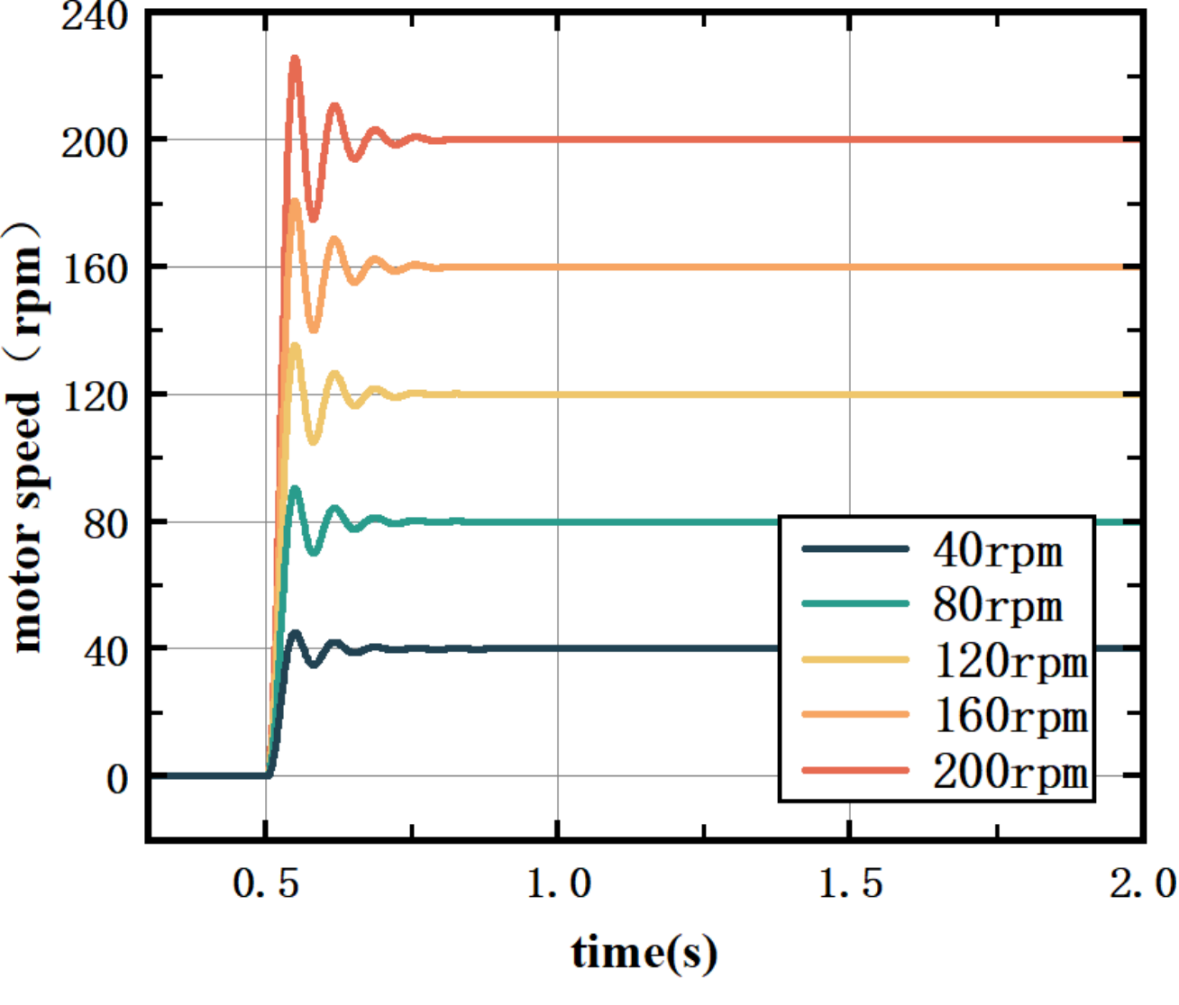
ATKB PID controller control effect.

**Fig. 11:**
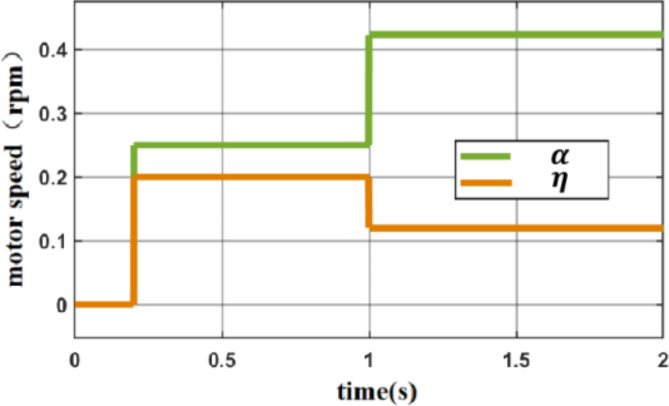
Learning rate and inertia coefficient variation with operating conditions.

In Fig. 10, the load of the motor is set to 500 NM, and the target speed of the motor is controlled at 40 rpm, 80 rpm, 120 rpm, 160 rpm, and 200 rpm. The ATKB-PID controller can regulate and stabilize the target speeds of the E1 motor of the stick mill. For different speeds, the ATKB-PID controller is stabilized within 0.3 s, and the PID parameters are successfully calibrated to suit the actual working conditions so that the algorithm can meet the needs of actual production. The learning rate and inertia coefficient of the BP algorithm of ATKB-PID with operating conditions are shown in Fig. 11.

Figure 10 shows the detailed data of each experiment, and the control effect is shown in Table 3.

**Table 3:** Test model results with different target speeds.

Motor speed	40 rpm	80 rpm	120 rpm	160 rpm	200 rpm
ess	0	0	0	0	0
tr	0.037 s	0.036 s	0.037 s	0.035 s	0.035 s
ts	0.282 s	0.284 s	0.275 s	0.278 s	0.292 s

According to Fig. 10; Table 2, there is not much difference in the control effect of the ATKB-PID controller when the target speed of the motor changes. The difference between the regulation time and the rise time is small, and the steady-state error is 0, which simultaneously satisfies the accuracy and rapidity requirements, and the control effect is significantly improved.

The range of conditions that may occur in more detailed group experiments for a 1450 production line during the hot rolling roughing process. The possible motor loads and motor target speeds are grouped. The possible ranges of motor load are divided into 200 NM, 400NM, 600NM, 800NM, and 1000NM, the possible ranges of motor target speed are divided into 40 rpm, 80 rpm, 120 rpm, 160 rpm, and 200 rpm, and the most important adjustment time is tested. The test results are shown in the Table 4.

**Table 4:** Test results of the vertical roller E1 motor under different working conditions.

Load/ Motor speed	40 rpm	80 rpm	120 rpm	160 rpm	200 rpm
200NM	0.276 s	0.272 s	0.280 s	0.275 s	0.266 s
400NM	0.280 s	0.282 s	0.274 s	0.278 s	0.255 s
600NM	0.276 s	0.280 s	0.275 s	0.278 s	0.284 s
800NM	0.268 s	0.266 s	0.268 s	0.272 s	0.326 s
1000NM	0.269 s	0.274 s	0.368 s	0.285 s	0.382 s

When the load of the E1 motor of the vertical roller mill is 1000 NM, the target speed is 120 rpm, the load is 1000 NM, the target speed is 200 rpm, the load is 800 NM, the target speed is 200 rpm, and the control effect of the ATKB-PID controller is poor. The regulation times are 0.368 s, 0.382 s and 0.326 s, respectively, and the regulation time under other conditions is less than 0.3 s. The growth of the regulation time due to the different sizes of motor loads is not obvious, so the ATKB-PID controller has a good control effect. The control effect of the above three samples is generally due to the faster load and motor target speed, which are located in the hot rolling roughing vertical roller mill E1 motor normal load and target speed range of the edge of the value, which rarely occur. This also led to the fact that when we designed the data set used by KNN, the data contained less extreme conditions in the production process, which led to the deviation of KNN’s prediction of controller parameters in the experiment, resulting in poor control effect. This is a possible future improvement direction to address extreme case parameter prediction while maintaining model complexity and considering industrial applications.

To sum up, the ATKB-PID controller proposed in this paper achieves the expected results and is able to satisfy both the rapidity and accuracy requirements to solve the micro tension control problem of hot strip roughing mills.

## Discussion

1. This paper proposes a micro tension control method for the roughing process that features a small steady-state error, short rise time, and short regulation time. The method’s accuracy, speed, and feasibility are verified through experiments.
2. The ATKB-PID controller proposed in this paper integrates a linear attention mechanism and adjusts the inertia and learning coefficients of the BP-PID controller using the KNN algorithm. It also designs an objective function to meet the complex requirements of micro tension control in the roughing process of hot strip milling.
3. In this paper, for hot rolling roughing mill E1 motors, the experimental range exceeds the actual working range, and the ATKB-PID controller still maintains a good control effect.
4. In this study, one of the main challenge we faced was how to maintain control effectiveness while reducing computational costs and simplifying the implementation process. We achieved this by employing a structurally simpler Linear-attention and designing it to allow for a smaller range of values for K, thereby reducing computational costs and the difficulty of its practical application.
5. Since actual production seldom experiences motor target speeds and loads near the maximum values of the set range, the ATKB-PID controller’s performance at these limits is not optimal. However, there is potential for improvement in these scenarios.

## Data Availability

The data that support the findings of this study are available on request from the corresponding author, upon reasonable request.
